# Letter to the Editor regarding the Article “Comparison of Follow-Up Length-Matched Single-Center Myelomeningocele Postnatal Closure Cohort to the Management of Myelomeningocele Study (MOMS) Trial Results”

**DOI:** 10.1159/000529013

**Published:** 2023-01-10

**Authors:** Stephanie Greene, Jasmine L. Hect, Kristin Weaver, Michael M. McDowell

**Affiliations:** ^a^Department of Neurosurgery, Children's Hospital of Pittsburgh University of Pittsburgh Medical Center, Pittsburgh, Pennsylvania, USA; ^b^Department of Neurosurgery, University of Mississippi Medical Center, Jackson, Mississippi, USA

Dear Editor,

In the interim between submission and publication of our study “Comparison of Follow-Up Length-Matched Single-Center Myelomeningocele Postnatal Closure Cohort to the Management of Myelomeningocele Study (MOMS) Trial Results” [1], it has come to our attention that several key follow-up studies have now been made available which are worthy of re-analysis. We appreciate the opportunity to present updates, taking into account recently published outcomes of the Management of Myelomeningocele Study (MOMS) [2, 3] to our analysis originally published in *Pediatric Neurosurgery* titled “Comparison of Follow-Up Length-Matched Single-Center Myelomeningocele Postnatal Closure Cohort to the Management of Myelomeningocele Study (MOMS) Trial Results” [1]. We acknowledge the limitations inherent in comparing data between retrospective cohort and randomized trial study designs as delineated in Dr. Houtrow's recent letter to the editor [4]. However, as is clearly outlined in our original manuscript, it was never the intent of the manuscript to imply that our results superseded results acquired through prospective analysis or that our results were inherently superior, and thus, the use of prenatal closure was invalid. Rather, we used our cohort to highlight our continued concerns about the durability of some of the outcomes of prenatal closure and the need to consider the risk-benefit balance for both mother and child. In this report, we present new analysis incorporating these recently published follow-up outcomes in the MOMS prenatal and postnatal repair arms (MOMS2), specifically for long-term ambulatory ability and rates of detethering procedures, in order to further the discussion of our aforementioned concerns. We voice our concerns not because we do not support prenatal closure, but in fact because we believe that the highest level of scrutiny will ultimately ensure its continued viability as part of the surgical armamentarium.

Analyses comparing long-term functional and surgical outcomes between the Pittsburgh cohort (average 10 years, range 2–22 years) and MOMS2 trial (average 7.9, range 5.9–10.3 years) are summarized in Figure 1 (upper panel) and reported in detail in Table 1. A total of 161 of the original 183 MOMS patients participated in MOMS2; reasons for the 12% attrition rate included 8 deaths (5 prenatal and 3 postnatal) and 14 patients lost to follow-up [2]. At long-term follow-up, prenatal closure resulted in a 27% rate of detethering compared to 19.6% in our cohort (*p* = 0.09), which trended toward significance despite the longer follow-up in our cohort. Table 2 summarizes within-cohort comparisons of short- and long-term functional and surgical outcomes for our Pittsburgh UPMC cohort and both arms of the MOMS trial. Houtrow and colleagues (2020) report a 3.8-fold increase in detethering rates in the prenatal closure arm during the additional follow-up period of MOMS2. Specifically, MOMS reports an 8% detethering rate at initial follow-up and 27% at 8-year follow-up (MOMS2) (*p* < 0.001). This is in contrast to our postnatal closure cohort, which saw a 10-fold increase across 10 years of follow-up. Refer to Figure 1 (lower panel) for summary of detethering incidence and ambulatory ability across follow-up within UPMC cohort and MOMS trial arms. Comparison of detethering rates between the MOMS prenatal and postnatal closure arms at longest available follow-up demonstrates that the prenatal closure saw a detethering rate of 27% compared to 15% for postnatal closure patients (*p* = 0.026; Table 1) [2]. Patients in the MOMS postnatal closure arm also experienced an increase in detethering incidence across follow-up from 1% to 15% (*p* = 0.002). Additionally, while fetoscopic repair has been explored as an even less invasive alternative to open fetal repair, the technical and dexterity limitations of this approach may be associated with even higher rates of problem at the repair site (e.g., cerebrospinal fluid leak, inclusion cyst, increased detethering) [5, 6].

Further, we provide comparison of ambulatory status at long-term follow-up between cohorts (Table 1) and comparison of within-cohort change across follow-up (Table 2). At long-term follow-up, MOMS prenatal closure patients ambulated independently in 29% of cases, which was found to not be significantly greater when compared to the 21% of independently ambulating cases in our postnatal closure cohort (*p* = 0.19). However, prenatal closure patients were more likely to walk with or without assistive devices (93%) compared to our cohort (65.8%) at longest follow-up (Table 1; any ambulation; *p* < 0.001). Importantly, while the prenatal closure arm saw an increase from 77.1% to 93% of patients ambulating with or without assistive devices across follow-up (*p* = 0.008), compared to published short-term follow-up of ambulatory status published by Houtrow and colleagues (2021) [3], the rate of independent ambulation in the prenatal closure group had dramatically decreased from 61.3% to 29% (*p* < 0.001; Tables 1, 2). Yet overall independent ambulation remained significantly greater in prenatal closure (29%) than postnatal closure cohort (11%) at longest follow-up (*p* = 0.006).

While the overall ambulatory function of the cohort of those with follow-up in the postnatal cohort is commendable, the low rate of independent ambulation coupled with the substantial rate of cord tethering continues to underscore our manuscript's concerns about the durability of ambulatory ability achieved with prenatal closure. Ultimately, the ability to compare a retrospective cohort to a prospective cohort such as MOMS and MOMS2 is limited by the differences in the data available, but it is our feeling that the MOMS2 data support many of the conclusions and concerns cited in our original manuscript. We remain optimistic, but cautious, about the risks and benefits for fetal myelomeningocele surgery as well as potential other intrauterine procedures in the future.

## Conflict of Interest Statement

The authors have no personal or institutional conflicts of interest with regard to the authorship and/or publication of this manuscript.

## Funding Sources

This research received no specific grant from any funding agency in the public, commercial, or not-for-profit sectors.

## Author Contributions

M.M and S.G were involved in the design and conception of this report. J.H. performed data analysis, compiled figures and tables, and drafted the primary manuscript. M.M, K.W., and S.G. critically revised the manuscript. All authors have approved the manuscript as it is written.

## Figures and Tables

**Fig. 1:**
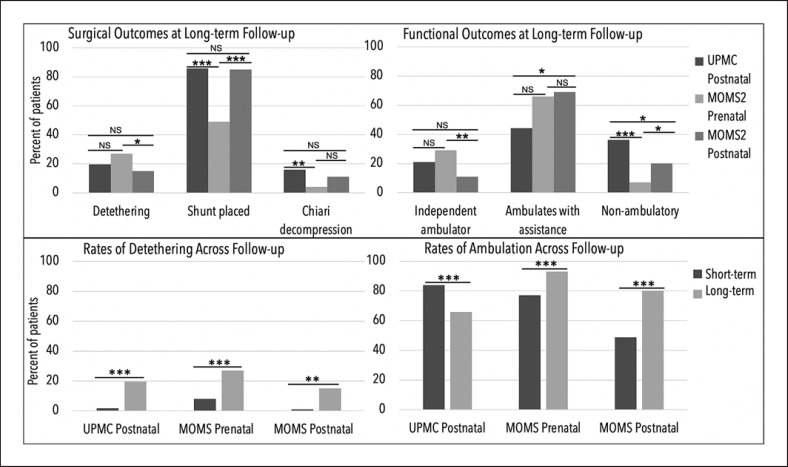
Top panel shows comparison across surgical and functional outcomes at longest follow-up across cohorts and MOMS trial arms. The bottom panel summarizes difference in rates of detethering and ambulatory status, defined as ambulation with or without assistance, across short-term follow-up (12-months) and long-term follow-up (10 years for UPMC and 8 years for MOMS) within the UPMC cohort and MOMS trial arms. *p < 0.05, **p < 0.01, ***p < 0.001.

**Table 1:** Comparison of long-term functional and surgical outcomes between Pittsburgh and MOMS cohorts

	UPMC postnatal cohort	MOMS2 prenatal cohort	UPMC versus MOMS2 prenatal, *p* value	MOMS2 postnatal cohort	UPMC versus MOMS2 postnatal, *p* value	MOMS2 prenatal versus postnatal, *p* value
Age at follow-up, years, M (SD)	10 (6.3)	7.8 (1.3)	<0.001	7.9 (1.2)	<0.001	
Shunt placed	134/156 (85.9)	38/78 (49)	**<0.001**	70/82 (85)	0.89	**<0.001**
Chiari	26/163 (16.0)	3/79 (4)	**0.007**	9/82 (11)	0.31	0.083
Detethering	32/163 (19.6)	23/79 (27)	0.09	12/82 (15)	0.33	**0.026**
Any ambulation	100/152 (65.8)	68/73 (93)	**<0.001**	59/74 (80)	**0.014**	**0.018**
Ambulatory status			**<0.001**		**0.001**	**0.004**
Independent	32/152 (21.1)	21/73 (29)	0.19	8/74 (11)	0.061	**0.006**
With assistance^a^	67/152 (44.1)	47/73 (66)	0.06	51/74 (69)	**0.011**	0.56
None	55/152 (36.2)	5/73 (7)	**<0.001**	15/74 (20)	**0.017**	**0.018**

a Includes “walking with orthotics only” and “walking with assistive devices” as reported in the study by Houtrow et al. [1] 2020 (Table 3).

**Table 2:** Within-cohort comparisons of short- and long-term outcomes

	UPMC postnatal 12-month/ 30-month^a^ follow-up	UPMC postnatal 10-year follow-up	p value	MOMS prenatal 12-month follow-up	MOMS2 prenatal 8-year follow-up	p value	MOMS postnatal 12-month follow-up	MOMS2 postnatal 8-year follow-up	p value
Shunt placed	134/156 (85.9)	134/156 (85.9)	0.79	31/78 (40)	38/78 (49)	0.26	66/80 (82)	70/82 (85)	0.62
Chiari	17/163 (10.4)	26/163 (16.0)	0.11	1/77 (1)	3/79 (4)	0.32	4/80 (5)	9/82 (11)	0.16
Detethering	3/163 (1.8)	32/163 (19.6)	<0.001	6/77 (8)	23/79 (27)	<0.001	1/80 (1)	12/82 (15)	0.002
Any ambulation Ambulation	129/154 (83.8)	100/152 (65.8)	<0.001	59 (77.1)	68/73 (93)	0.008	38/78 (48.7)	59/74 (80)	0.003
Independent	39/154 (25.3)	32/152 (21.1)	0.36	47/76 (61.3)	21/73 (29)	<0.001	25/78 (32.1)	8/74 (11)	<0.001
With assistance^1^	90/154 (58.4)	67/152 (44.1)	0.19	12/76 (15.8)	47/73 (66)	<0.001	13/78 (16.7)	51/74 (69)	<0.001
None	25/154 (16.3)	55/152 (36.2)	<0.001	17/76 (22.4)	5/73 (7)	0.008	40/78 (51.3)	15/74 (20)	<0.001

a Short-term surgical outcomes were measured at 12 months and functional outcomes at 30 months, as reported in the study by Weaver et al. [1] 2021 (Tables 2 and 3).

1 Includes “walking with orthotics only” and “walking with assistive devices” as reported in the study by Houtrow et al. [2] 2020 (Table 3).
